# Infant Formula with 50% or More of Palmitic Acid Bound to the sn-2 Position of Triacylglycerols Eliminate the Association between Formula-Feeding and the Increase of Fecal Palmitic Acid Levels in Newborns: An Exploratory Study

**DOI:** 10.3390/nu16111558

**Published:** 2024-05-21

**Authors:** Hiromichi Shoji, Hiroko Arai, Satsuki Kakiuchi, Atsushi Ito, Keigo Sato, Shinji Jinno, Naoto Takahashi, Kenichi Masumoto, Hitoshi Yoda, Toshiaki Shimizu

**Affiliations:** 1 Department of Pediatrics, Faculty of Medicine, Juntendo University, 3-1-3, Hongo, Bunkyo-ku, Tokyo 113-8431, Japan; 2 Department of Neonatology, Faculty of Medicine, Toho University, 6-11-1, Omorinishi, Ota-ku, Tokyo 143-8541, Japan; kenichi.masumoto@med.toho-u.ac.jp (K.M.); yoda@med.toho-u.ac.jp (H.Y.); 3 Department of Pediatrics, The University of Tokyo, 7-3-1, Hongo, Bunkyo-ku, Tokyo 113-8655, Japan; kakiuchi.satsuki@twmu.ac.jp (S.K.); neonotamago@gmail.com (A.I.); ntakahashi@m.u-tokyo.ac.jp (N.T.); 4 Food Microbiology and Function Research Laboratory, Meiji Co., Ltd., 1-29-1, Nanakuni, Hachioji, Tokyo 192-0919, Japan; keigo.satou@meiji.com (K.S.); shinji.jinno@meiji.com (S.J.); 5 Wellness Science Labs, Meiji Holdings Co., Ltd., 1-29-1, Nanakuni, Hachioji, Tokyo 192-0919, Japan; 6 Department of Pediatrics and Adolescent Medicine, Graduate School of Medicine, Juntendo University, 3-1-3, Hongo, Bunkyo-ku, Tokyo 113-8431, Japan; tshimizu@juntendo.ac.jp

**Keywords:** sn-2 palmitate, infant formula, breast milk

## Abstract

The binding ratio of palmitic acid (PA) at the sn-2 position of triacylglycerols in infant formulas is lower than that in breast milk, resulting in higher levels of fecal PA. Even if the ratio is increased to 40–50%, fecal PA levels in formula-fed infants remain higher than those in breast–fed infants. In Japan, infant formulas with 50% or more of PA bound to sn-2 (high sn-2 PA milk) are commercially available; however, their effects on PA excretion have not been investigated. Therefore, this observational study aimed to preliminarily evaluate whether the feeding volume of high sn-2 PA milk is significantly associated with fecal total/soaped PA levels in newborns. Infant formulas were classified as high (≥50% of PA bound to sn-2) or low sn-2 (<50%) PA milk. Associations between feeding volume of high or low sn-2 PA milk and fecal PA levels were evaluated using multiple regression analysis models. The results showed that the feeding volume of low sn-2 PA milk was positively associated with fecal total/soaped PA levels, while there was no significant association between those of high sn-2 PA milk and fecal total/soaped PA levels. Our preliminary study suggests that high sn-2 PA milk may reduce increased fecal PA levels in formula-fed newborns.

## 1. Introduction

Breast milk is an essential nutrient source for the healthy growth and development of infants. Triacylglycerols (TGs) are a major component of the fat in breast milk and provide approximately 50% of the energy [1]. Palmitic acid (PA) accounts for approximately 20% of the total fatty acids in TGs [2]. Approximately 70% of PA in breast milk selectively binds to the stereospecifically numbered sn-2 position of glycerol [3,4,5,6,7]. Since intestinal lipase selectively cleaves the binding sites at sn-1 and sn-3 of glycerol, PA bound at sn-2 is absorbed as monoacylglycerols, which creates micelles with biliary acids, allowing it to be absorbed quickly [3]. Free PA from sn-1 and sn-3 positions is water-insoluble due to its high melting point, causing it to form soaped PA with minerals, which is unfavorable for absorption. Therefore, PA in breast milk is thought to preferentially bind to sn-2 to help its absorption. The binding ratio of PA at sn-2 is highly beneficial for improving the nutritional performance of breast milk substitutes.

Increasing the binding ratio of PA at sn-2 in infant formulas has various benefits. In general, infant formulas are composed of vegetable oil, which has a low binding ratio of PA at sn-2 (approximately 10%) [8]. Therefore, formula-fed infants have higher excretion of free and soaped PA in stools than breastfed infants. Several researchers have reported that increasing the binding ratio of PA at sn-2 reduces fecal total and soaped PA in formula-fed infants [9,10,11,12,13,14,15,16,17,18]. By preventing the formation of soaped PA in the intestinal tract, high sn-2 PA provides several benefits for infants, such as greater bifidogenicity in the intestinal tract [19,20], softer stools [9,18,21], increased mineral absorption [9,11,12,18,22], and enhanced bone maturation [13,21,23]. To the best of our knowledge, studies on the impact of increasing the binding ratio of PA at sn-2 in infant formula are limited [24]; however, increasing this ratio could improve the absorption of PA and the intestinal environment.

Studies comparing the effects of infant formula with 50% or more of PA binding at sn-2 on PA excretion in stools are limited. Several studies using study formulas with up to 50% of PA bound to sn-2 have shown that excretion of PA in the stools of study formula-fed infants is significantly reduced compared with the stools of control formula-fed infants. However, there was still significantly higher PA excretion in stools in study formula-fed infants than in breastfed infants [13,14,16,17]. Although infant formulas with 50% or more of PA bound to sn-2 are commercially available in Japan, there is no report of an association between these formulas (with ≥50% of PA bound to sn-2)-fed infants and their PA excretion in stool. Therefore, this postnatal observational study of 1-month-old infants preliminarily evaluated whether the feeding volume of infant formulas with 50% or more of PA bound to sn-2 is associated with an increase in total PA and soaped PA levels in stools.

## 2. Materials and Methods

### 2.1. Categorization of Infant Formulas

Among commercially available infant formulas in Japan, those with 50% or more of PA bound to sn-2 were categorized as high sn-2 PA milk, and those with less than 50% were categorized as low sn-2 PA milk (Table 1). The percentages of PA in total fat, fat contents, and energy contents of infant formula and breast milk are shown in Table S1. The methods for measuring each nutrient are shown in the Supplementary Materials.

### 2.2. Study Design and Participants

This multicenter observational study was conducted in Tokyo, Japan. Clinical visits were scheduled at 1 month. All legal guardians of each infant provided informed consent before participating in the study. The study was conducted in accordance with the Declaration of Helsinki; the protocol was approved by the ethics committee of Juntendo University Hospital (approval numbers: 17-313 and 20-333, approval dates: 30 March 2018 and 7 January 2021), the Research Ethics Committee of the University of Tokyo (approval numbers: 2018011NI-(1) and 2020354NI, approval dates: 13 September 2018 and 17 February 2021), and the ethics committee of Toho University Omori Medical Center (approval numbers: M18030 and M20327, approval dates: 21 June 2018 and 1 May 2021).

Healthy mother-infant pairs, whose infants were born at one of the three centers, were eligible for participation. Infants who were twins or more, had a critical infectious disease, metabolic disease, or severe congenital abnormality, or were deemed inappropriate by the physician or research physician, were excluded from the study. Infant-mother pairs who provided informed consent were enrolled between September 2018 and September 2019 and between January 2021 and June 2022. The inclusion criteria included infants who were born at term (gestational age ≥37 weeks, <42 weeks), had a normal birth weight (≥2500 g, <4000 g), and had available fed-formula data. In this study, the power calculation of the analysis was not performed because this study was conducted as a preliminary evaluation.

### 2.3. Data and Sample Collection

Demographic data were collected using questionnaires answered by the mothers. The data collected at enrollment were as follows: age of the mothers at birth, weight before pregnancy, maximum weight during pregnancy, nationality of mother and father, parity, caesarian section (C-section), administration of antimicrobials to the mother before birth, intake of supplements during pregnancy, infant’s sex, date of birth, gestational age, infant’s weight, height, and head circumference at birth.

Feeding data were collected using questionnaires. Mothers filled in the brand name of infant formula that they fed their infants, and data on the feeding volume per day from 1 week prior to the day of the 1-month clinical visit were collected. Data on the administration of antimicrobials to infants until 1 month was also collected using questionnaires at the 1-month clinical visit.

Stool and breast milk samples were collected at the participants’ homes before the day of the 1-month clinical visit. Participants were instructed to collect stool excreted in diapers and to collect breast milk after the infant had finished feeding in the morning. We instructed participants to freeze each biological sample in a home freezer after collection and to use cooling agents when transporting them from home to the clinic on the day of the 1-month clinical visit. In the clinic, medical staff collected samples from the mothers, which were stored at –80 °C until analysis.

In subsequent data tabulations (demographic data, anthropometric data, and stool analysis), infants were sub-grouped into exclusively breastfed, high sn-2 PA milk-fed, and low sn-2 PA milk-fed groups. Exclusively breastfed infants were defined as those who were not recorded to have been fed infant formulas. The high sn-2 PA milk-fed sub-group was defined as those who were fed more of the high sn-2 PA milk than the low sn-2 PA milk. The low sn-2 PA milk-fed sub-group was defined as those who were fed more of the low sn-2 PA milk than high sn-2 PA milk. All feeding types were determined using data from 1 week prior to the 1-month clinical visit. Feeding volume was calculated as mL/day/body weight.

### 2.4. Anthropometric Data Collection

Data on infants’ weight, length, and head circumference at the 1-month clinical visit after birth were collected by mothers by transcribing them onto a questionnaire from their mother-child handbooks. The date of the 1-month clinical visit was also collected. The age in days at the 1-month clinical visit was calculated by subtracting the date of birth from the date of the 1-month clinical visit.

### 2.5. Analysis of Total PA and Soaped PA Levels in Stool

The frozen stool was thawed at room temperature. Stool pH was measured using a tester (pH Spear; Eutech, Singapore). After pH measurements, the stool was inactivated by heating at 98 °C for 30 min and stored at −20 °C until analysis.

The stool was lyophilized prior to fatty acid analysis. Lipids were extracted from less than 100 mg of dried stools. The total PA and non-soaped PA contents were individually measured for each stool sample. The extraction solvent used was an upper layer of heptane:diethylether:ethanol (1:1:1). For non-soaped PA measurements, the extraction solvent was directly added to the stool and mixed thoroughly. The solvents were centrifuged at 1940× *g* and 4 °C for 10 min, and the upper layer was collected. The extraction process was repeated twice. All the collected solvents were dried under flowing nitrogen. Dried lipids were dissolved in hexane. The lipid extracts were obtained by dehydration with anhydrous sodium sulfate. Lipid contents were measured using the dry weight of the extracts. For total PA measurements, hydrochloric acid was added to convert the soaped fatty acids to free fatty acids before the addition of the extraction solvent. The following procedures were the same as for the non-soaped PA measurements.

For the quantification of PA in the gas chromatography analysis, 4 mg of the lipid extract was used. As an internal standard, 1 mg of margaric acid was added to the lipids. Next, lipids were methylated using a fatty acid methylation kit (nacalai tesque). Gas chromatography analysis was performed using a GC-2010 Plus (Shimadzu, Kyoto, Japan) coupled with a flame ionization detector. Separation was achieved using a ZB-FAME column (length = 20 m, internal diameter = 0.18 mm, film thickness = 0.15 µm; Phenomenex, Torrance, CA, USA). The conditions for analysis were as follows: career gas, helium; total flow rate, 36.2 mL/min; column flow rate, 0.61 mL/min; and column temperature, 100–100 °C/0–2 min, 100–140 °C/2–6 min, 140–190 °C/6–23 min, 190–260 °C/23–25 min, and 260–260 °C/25–27 min. The PA contents in each sample were calculated using the peak area ratio of PA and the internal standard. Soaped PA was defined as non-soaped PA subtracted from the total PA. The total PA and soaped PA level was calculated as the PA amount per used dried stool for the analysis (mg/g-day-stool). If the calculated soaped PA level was negative, it was set to 0 mg/g dry stool.

### 2.6. Statistical Analysis

To evaluate the association between feeding volume of high sn-2 PA milk or low sn-2 PA milk and infants’ weight, height, or head circumference at 1-month, multiple regression models using feeding volume of high sn-2 PA milk and low sn-2 PA milk as explanatory variables were conducted. The feeding volume of high sn-2 PA milk or low sn-2 PA milk was calculated as the average feeding volume from 1 week to 1 day before the 1-month clinical visit day per weight at the 1-month period. We evaluated the association based on the partial regression coefficient (β) and 95% confidential interval (95%CI) of feeding volume of high sn-2 PA milk and low sn-2 PA milk. In the multiple regression model of total or soaped PA in stool, gestational age at birth, infants’ sex, age in days at the 1-month clinical visit, and C-section birth were considered confounders.

To evaluate the association between the feeding volume of high sn-2 PA milk or low sn-2 PA milk and total PA, soaped PA, or pH in stool, multiple regression models employing feeding volumes of high sn-2 PA milk and low sn-2 PA milk as explanatory variables were conducted. We evaluated the association based on the β and 95% CI of feeding volumes of high sn-2 PA milk and low sn-2 PA milk. In the multiple regression model of total or soaped PA, pH in stool was considered a confounder because saponification was deviated by the acid. None of the other confounders were considered in the stool pH evaluation.

Mother-infant pairs with missing data were excluded from each statistical analysis. Statistical significance was set at a *p*-value of less than 0.05. In multiple regression models, the 95%CI of β not crossing 0 was considered statistically significant. All statistical analyses were conducted using the R statistical software (version 4.1.3) and BellCurve for Excel (version 3.20).

## 3. Results

### 3.1. Study Population and Demographic Data

**Figure 1:**
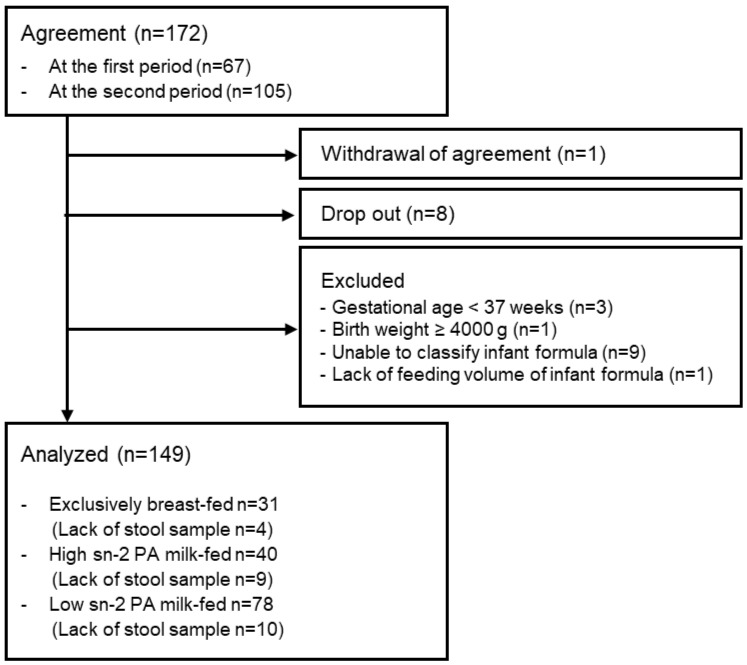
Flowchart of participants. Abbreviations: sn, stereospecific numbering; PA, palmitic acid.

The flowchart of the participants in this study is shown in Figure 1, and their demographic data are shown in Table 2. A total of 31 infants were exclusively breastfed, 40 were exclusively or partially fed high sn-2 PA milk, and 78 were exclusively or partially fed low sn-2 PA milk. There were no significant differences between sub-groups regarding participant demographic data.

The binding ratio of PA at sn-2 in the breast milk of the participants was 73.9 ± 6.1% (n = 139) (Table S1). Out of 149 participants, 140 breast milk samples were collected. However, one sample had insufficient volume to measure the sn-2 ratio of PA.

### 3.2. Association between Feeding Volume of High or Low sn-2 PA Milk and Anthropometric Data

The data on the growth of infants in this study from the first month of birth are summarized in Table 3. There were no significant differences between the sub-groups.

**Figure 2:**
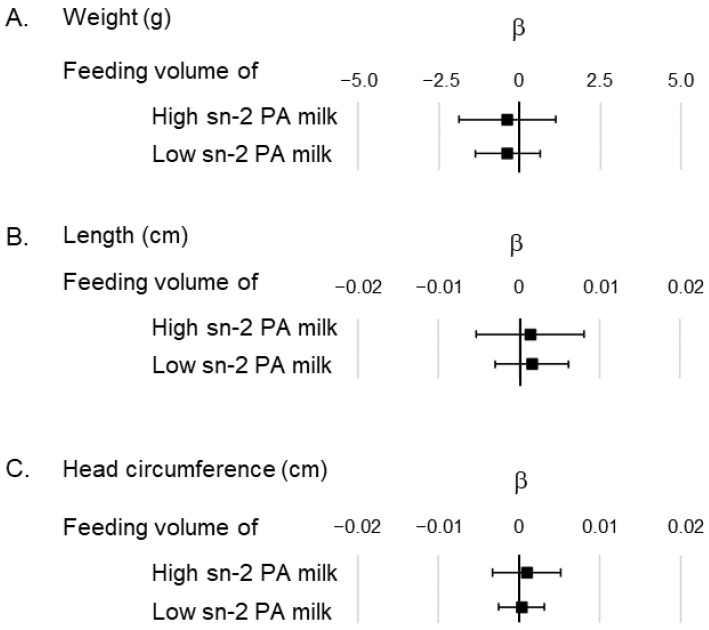
Association of feeding volume of high and low sn-2 PA milk in the multiple regression model showing the anthropometric data. (**A**) Association between feeding volume of high/low sn-2 PA milk and the infants’ weight; (**B**) Association between feeding volume of high/low sn-2 PA milk and the infants’ length; (**C**) Association between feeding volume of high/low sn-2 PA milk and the infants’ head circumference. All analysis models were adjusted by gestational week at birth, sex of the infant, age in days at the 1-month clinical visit, and caesarian section. The unit of feeding volume is expressed as daily feeding volume by body weight (mL/day/bw kg). β represents the partial regression coefficient. ■ represents the partial regression coefficient for each factor, and the bar indicates the 95% confidence interval of β. Abbreviations: sn, stereospecific numbering; PA, palmitic acid.

Next, we conducted a multiple regression model based on the weight, height, and head circumference of the infants at 1 month, which was related to the feeding volumes of the high and low sn-2 PA milk (Figure 2); however, the analysis showed no significant associations between the two.

### 3.3. Association between Feeding Volume of High or Low sn-2 PA Milk and Fecal PA Levels

**Figure 3:**
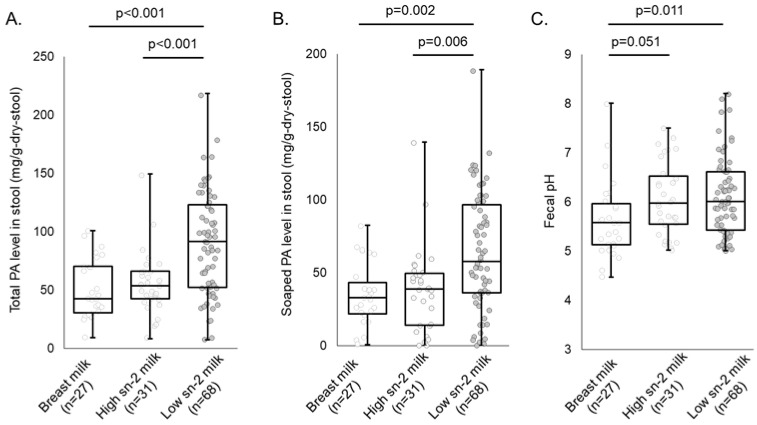
Results of total and soaped PA levels in stool and fecal pH analysis. The Steel–Dwass test was performed to compare each fecal indicator level in each sub-group. (**A**) Total PA level in stool; (**B**) Soaped PA level in stool; (**C**) Fecal pH. The statistical results with *p* < 0.1 are shown on the graph. Abbreviations: sn, stereospecific numbering; PA, palmitic acid.

Figure 3 shows the total and soaped PA levels in stool and fecal pH at 1 month of age. There was no significant difference in the feeding volume between infants who were fed high or low sn-2 PA milk (Table 2). In contrast, the total and soaped PA levels in the stool of infants fed low sn-2 PA milk were significantly higher than those of infants fed high sn-2 PA milk and exclusively breastfed infants (Figure 3A,B). There were no significant differences in the total and soaped PA levels in stool between infants fed high sn-2 PA milk and exclusively breastfed infants (Figure 3A,B). Fecal pH was significantly higher in infants fed low sn-2 PA milk than in breastfed infants (Figure 3C). The results of stool Ca levels, stool water levels, and stool consistency in each sub-group are shown in Table S2.

**Figure 4:**
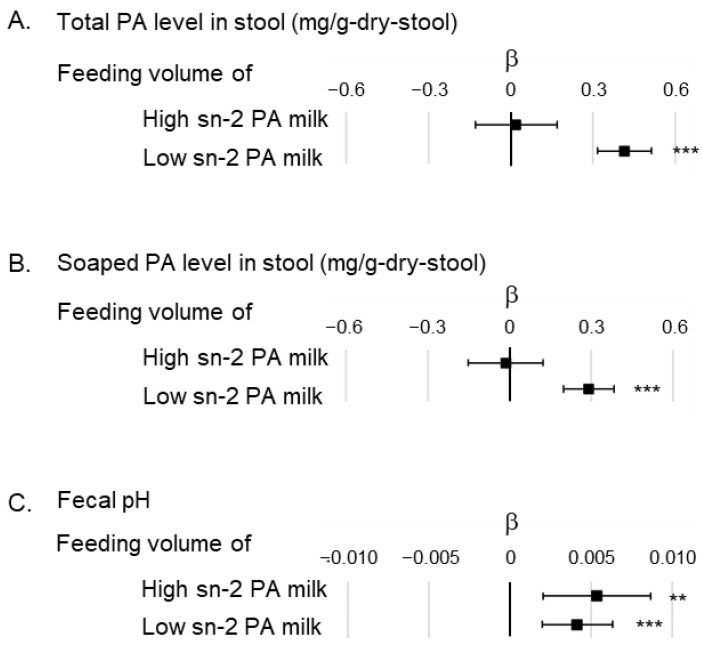
Association between feeding volume of high and low sn-2 PA milk in the multiple regression model showing total and soaped PA levels in stool, and fecal pH. (**A**) Correlation between feeding volume of high or low sn-2 PA milk and total PA level in stool. The analytical model is adjusted by fecal pH; (**B**) association between feeding volume of high or low sn-2 PA milk and soaped PA level in stool. The analytical model is adjusted by fecal pH; (**C**) association between feeding volume of high or low sn-2 PA milk and fecal pH. No confounder was adjusted in this analytical model. The unit for feeding indicates the daily volume of milk per body weight (mL/day/kg). β indicates the partial regression coefficient. ■ indicates the partial regression coefficient of each factor, and the bar indicates the 95% confidence interval of β. ** *p* < 0.01, *** *p* < 0.001. Abbreviations: sn, stereospecific numbering; PA, palmitic acid.

Multiple regression analysis was performed to determine whether total and soaped PA levels and fecal pH in the first month were associated with the feeding volume of the high or low sn-2 PA milk (Figure 4). The analysis showed a significant positive association between total or soaped PA levels and the feeding volume of low sn-2 PA milk (β: 0.42; 95%CI: 0.32–0.51 [total PA level in stool], β: 0.29; 95%CI: 0.20–0.38 [soaped PA level in stool]), but no significant association between high sn-2 PA milk and feeding volume (β: 0.02; 95%CI: −0.13–0.17 [total PA level in stool], β: −0.01; 95%CI: −0.15–0.12 [soaped PA level in stool]). Fecal pH was positively associated with feeding volume of both types of infant formula (β: 0.0054; 95%CI: 0.0020–0.0087 [feeding volume of high sn-2 PA milk], β: 0.0042; 95%CI: 0.0020–0.0063 [feeding volume of low sn-2 PA milk]).

## 4. Discussion

As infant formulas with various levels of PA binding ratios at sn-2 are commercially available in Japan, we investigated associations between the feeding volume of each infant formula and fecal PA levels in 1-month-old infants in this observational study. We found that feeding infants with formulas with less than 50% of PA bound to sn-2 was positively associated with increased fecal total and soaped PA levels in infants in the first month of life, while no significant association was observed with those fed with formulas with 50% or more of PA bound to sn-2. This indicates that the fecal PA level of 1-month-old formula-fed infants and mixed-fed infants can be maintained at the same level as that of breastfed infants using infant formulas with 50% or more of PA bound to sn-2. Hence, our results provide further insights into improving infant formulas.

This study shows that infant formulas with 50% or more of PA bound to sn-2 can prevent increased fecal PA levels, which are usually observed in formula-fed infants. Several studies have reported that infant formulas with up to 50% of PA bound at sn-2 (study formula) lead to lower total and soaped PA excretion than infant formulas with less PA bound at sn-2 (approximately 10%) [10,11,12,13,14,15,16,17]. However, some of these reports show that significant differences are still observed between formula-fed and breastfed infants [13,14,16,17]. In these studies, the sn-2 ratio of PA in formula was less than 50%; however, the present study evaluated infant formula with 50% or more of PA bound to sn-2. Based on our results, infant formula with 50% or more of PA bound to sn-2 is essential to achieve almost equivalent fecal PA levels as breastfed infants in the first month of life.

Additionally, feeding infants with formulas with 50% or more of PA bound to sn-2 potentially enables the formation of a bifidobacteria-dominated gut microbiome via suppression of the production of soaped PA in the gut. Reducing soaped PA is considered a key factor in the formation of an intestinal microbiome similar to that of breastfed infants. Some studies have shown that formula-fed infants have an intestinal microbiome with fewer bifidobacteria than breastfed infants [25,26]. One report showed that soaped PA inhibits the growth of bifidobacteria [27]. Furthermore, infant formula fortified the sn-2 PA (approximately 40% in total PA) and increased bifidobacteria in infants’ gastrointestinal tracts [19,20]. Guo et al. [28] reported that high sn-2 palmitate changed the gut microbiome and fecal metabolome profile, potentially benefiting infants. Interestingly, the present study has shown that the feeding volume of infant formula with 50% or more of PA bound to sn-2 is not significantly associated with an increasing fecal soaped PA (Figure 4A). Thus, increasing the sn-2 bond ratio of PA to over 50% in infant formula potentially contributes more effectively to the inhibition of PA saponification in the gut, which is important for the formation of a bifidobacterium-dominated gut microbiota.

The positive association between feeding volume of infant formula and fecal pH observed in this study may be caused by the different nutritional characteristics of infant formulas from breast milk other than the ratio of PA bound at sn-2. In this study, feeding volume of both high and low sn-2 PA milk showed a positive association with fecal pH (Figure 4C). Therefore, common characteristics in both types of infant formula may be associated with this increased fecal pH. In general, infant formulas contain lower amounts of indigestible oligosaccharides (human milk oligosaccharides) [29,30] and higher alkali metal calcium levels [31] than breast milk. These common nutritional characteristics of commercially available infant formulas may contribute to a high fecal pH.

Further investigation is required to obtain infants’ outcomes associated with suppressed PA excretion. Suppression of PA excretion causes fluctuations in energy intake, which may influence the physical growth of infants. However, in this study, no association was observed between the feeding volumes of high or low sn-2 PA milk and physical growth up to 1 month after birth (Figure 2). Furthermore, several studies have shown that the binding ratio of PA at sn-2 in infant formula does not affect physical growth [12,13,15,16,17,18,32]. Hence, the effect of suppressing the amount of PA excreted in formula-fed infants may require the evaluation of outcomes other than physical growth.

This study suggests that the use of high sn-2 PA milk can maintain a gut environment similar to that of breastfed infants, including mixed-fed infants. As of 2015, in Japan, approximately 40–50% of infants are mixed-fed at 1 month after birth, and less than 5% are exclusively formula-fed [33]. In this study, 140 breast milk samples were collected from 149 participants, indicating that the majority (140 of 149; 94.0%) of infants are fed breast milk, at least partially. Therefore, it was important that the results were obtained on infants close to the actual population of feeding types in Japan. The exclusive or partial use of infant formula with 50% or more of PA bound at sn-2 may be a better alternative to present breast milk substitutes.

This study has several limitations due to its observational nature. First, the causal associations between the findings are unclear since this study was conducted as a preliminary evaluation with no intervention of high and low sn-2 PA milk feeding. These need to be investigated through interventional studies. Second, low sn-2 PA milk contains a wide range of sn-2 PA ratios (6.1–39.5%), which may prevent the detection of associations between low sn-2 PA milk feeding and fecal PA levels. However, we detected a positive association between low sn-2 PA milk feeding and fecal PA levels. Multiple regression models categorized by three formula feeding volumes (high: Formula A, B; mid: Formula C, D; low: E-J) showed a positive association between fecal PA levels and low and mid sn-2 PA milk feeding, while no significant association was found with high sn-2 PA milk feeding (Tables S3 and S4), supporting the accuracy of our analysis. Third, our multiple regression analysis did not include the PA sn-2 ratio of individual breast milk and breast milk feeding volume as explanatory variables. We found that the PA sn-2 ratio of individual breast milk was consistently high (approximately 70%) with low variation (Table S1), making it unlikely to strongly influence the multiple regression models. In addition, considering that breast milk feeding volume is expected to be negatively associated with the feeding volume of both high and low sn-2 PA milk, including breast milk feeding volume as a variable in the multiple regression analysis would be inappropriate. Fourth, there were differences in the composition of the high and low sn-2 PA milk other than the binding ratio of PA at sn-2. These differences might have confounded the associations observed in this study. Fifth, this study evaluated only the fecal PA level, and the amount of PA absorbed into the body was not examined.

## 5. Conclusions

Based on the present results, infant formulas with 50% or more of PA bound to sn-2 are not associated with increased fecal total and soaped PA levels in newborns. These infant formulas suppress total and soaped PA levels in stool, possibly resulting in a bifidobacterium-dominated gut microbiota and high mineral absorption in formula-fed infants, although this observational study was conducted as a preliminary evaluation. Further studies are needed to extensively examine the association between feeding with high sn-2 PA milk and outcomes.

## Figures and Tables

**Table 1:** Percentage of PA at the sn-2 position in infant formula available in Japan.

Infant Formula	Sn-2 PA in Total PA	Category
	(%)	
A	55.3	High sn-2 PA milk
B	52.9	
C	39.5	Low sn-2 PA milk
D	37.5	
E	11.9	
F	11.7	
G	11.4	
H	10.3	
I	6.4	
J	6.1	

Abbreviations: PA, palmitic acid; sn, stereospecific numbering.

**Table 2:** Characteristics of the study population.

	Unit	Total (n = 149)	Sub-Grouped by Feeding Types			
			Exclusively Breastfed Infants (n = 31)	Formula-Fed Infants		*p* Value *
				High sn-2 PA Milk-Fed (n = 40)	Low sn-2 PA Milk-Fed (n = 78)	
Maternal data						
Age at birth	days	34.4 ± 5.4	32.9 ± 4.8	34.5 ± 5.3	35.0 ± 5.5	0.172
BMI before pregnancy	kg/m^2^	21.5 ± 3.5	21.1 ± 3.0	21.5 ± 3.5	21.6 ± 3.7	0.816
Maximum BMI during pregnancy	kg/m^2^	25.5 ± 3.3	25.1 ± 2.7	25.6 ± 3.6	25.6 ± 3.4	0.749
Nationality (not Japanese)	person	2 (1.3%)	0 (0%)	1 (2.5%)	1 (1.3%)	0.667
Missing		1 (0.7%)	1 (3.2%)	0 (0%)	0 (0%)	
History of prior births before	person	69 (46.3%)	15 (48.4%)	17 (42.5%)	37 (47.4%)	0.849
C-section (yes)	person	47 (31.5%)	8 (25.8%)	14 (35.0%)	25 (32.1%)	0.704
Administration of antimicrobials before birth (yes)	person	25 (16.8%)	6 (19.4%)	6 (15.0%)	13 (16.7%)	0.856
Missing		1 (0.7%)	1 (3.2%)	0 (0%)	0 (0%)	
Intake of lactobacillus/bifidobacteria supplements during pregnancy (yes)	person	8 (5.4%)	1 (3.2%)	2 (5.0%)	5 (6.4%)	0.811
Missing		1 (0.7%)	1 (3.2%)	0 (0%)	0 (0%)	
Paternal data						
Nationality (not Japanese)	person	5 (3.4%)	1 (3.2%)	2 (5.0%)	2 (2.6%)	0.786
Missing		1 (0.7%)	1 (3.2%)	0 (0%)	0 (0%)	
Infant data						
Sex (girls)	person	80 (53.7%)	16 (51.6%)	24 (60.0%)	40 (51.3%)	0.665
Missing		1 (0.7%)	1 (3.2%)	0 (0%)	0 (0%)	
Gestational age at birth	weeks	39.3 ± 1.1	39.3 ± 0.9	39.3 ± 1.2	39.2 ± 1.2	0.928
Weight at birth	g	3058.5 ± 304.9	3059.7 ± 280.9	3070.6 ± 295.1	3051.7 ± 321.9	0.951
Length at birth	cm	48.7 ± 2.9	48.6 ± 1.5	48.6 ± 1.6	48.8 ± 3.8	0.907
Head circumference at birth	cm	33.6 ± 1.2	33.4 ± 1.2	33.7 ± 1.1	33.7 ± 1.2	0.452
Age at the 1-month clinical visit	days	33.6 ± 5.3	33.9 ± 5.6	33.9 ± 4.0	33.3 ± 5.7	0.790
Use of antimicrobials until 1 month (yes)	person	3 (2.0%)	0 (0%)	0 (0%)	3 (3.8%)	0.248
Feeding volume of infant formula	mL/day/kg	-	-	67.4 ± 57.3	84.7 ± 61.8	0.143

Abbreviations: sn, stereospecific numbering; C-section, caesarian section; BMI, body mass index. * One-way analysis of variance, Kruskal–Wallis test, or chi-square test.

**Table 3:** Results of anthropometric data collection.

		Formula-Fed		*p*-Value *
	Exclusively Breastfed	High sn-2 PA Milk-Fed	Low sn-2 PA Milk-Fed	
	(n = 31)	(n = 40)	(n = 78)	
At 1 month				
Weight (kg)	4.4 ± 0.5	4.3 ± 0.4	4.3 ± 0.4	0.630
Length (cm)	53.9 ± 1.7	53.9 ± 1.8	53.6 ± 1.9	0.629
Head circumference (cm)	37.0 ± 1.2	37.0 ± 1.3	37.0 ± 1.1	0.990

* One-way analysis of variance. Abbreviations: sn, stereospecific numbering; PA, palmitic acid.

## Data Availability

The data presented in this study are available on request from the corresponding author. The data are not publicly available due to restrictions of ethics.

## Supplementary Materials

The following supporting information can be downloaded at: https://www.mdpi.com/article/10.3390/nu16111558/s1, Supplementary information: Analysis of infant formula and breast milk. Supplementary Table S1: PA ratio in sn-2 position, PA ratio in total fat, and fat and energy content in breast milk at 1 month and infant formula. Table S2: Results of stool characteristics. Table S3: Association between feeding volume of high/mid/low sn-2 PA milk in the multiple regression analysis showing total PA levels in stool. Table S4: Association between feeding volume of high/mid/low sn-2 PA milk in the multiple regression analysis showing soaped PA levels in stool. References [34,35,36] are cited in Supplementary Materials.
