# Supplementary material for: Infant Formula with 50% or More of Palmitic Acid Bound to the sn-2 Position of Triacylglycerols Eliminate the Association between Formula-Feeding and the Increase of Fecal Palmitic Acid Levels in Newborns: An Exploratory Study
Source: Nutrients. 2024 May 21;16(11):1558. doi: 10.3390/nu16111558 (PMC11173637; doi:10.3390/nu16111558)
Supplement: Supplementary file 1 [file nutrients-16-01558-s001.zip › nutrients-2997826-supplementary.pdf]

Supplementary materials

Supplementary information

Analysis of infant formula and breast milk

Prior to analysis, breast milk was inactivated by heating at 100 °C for 30 min and stored at –20 °C. Breast milk was thawed in flowing water and sonicated at 40 °C for 10 min before analysis. The fat content of the breast milk was evaluated using a decompression drying method. The energy contributions of fat, protein, carbohydrate, ash, and solid content were calculated. Protein contents were estimated using the Dumas' method for the quantitative determination of nitrogen. Ash was measured using the direct ashing method. The solid content was determined using the atmospheric heat-drying method. The carbohydrate content was determined by subtracting the solid contents of fat, protein, and ash. The calorie contribution is as follows: Fat, 9 kcal/g; protein, 4 kcal/g; and carbohydrate, 4 kcal/g. The infant formula was prepared according to the manufacturer's instructions before analysis. The fat content and energy of the infant formula were calculated from the product labeling.

The procedure of analysis of the sn-2 palmitic acid (PA) bonding ratio in infant formula and breast milk was performed according to Watanabe et al. [1]; the procedure is briefly described below. After sample preparation, 3 mL of breast milk or infant formula was used for lipid extraction. Lipid extraction was performed using the Bligh and Dyer method [2]. Briefly, chloroform:methanol (2:1 [v/v]) was added to the breast milk or infant formula. After mixing, a 0.7% (w/v) sodium chloride solution was added to the solvent and mixed thoroughly. Next, centrifugation was performed, and the lower layer was collected and dehydrated using anhydrous sodium sulfate. The extracted lipids were dissolved in chloroform after evaporation. The lipid content was measured as the dry weight of the extracts. If insufficient amounts of lipids were obtained for the subsequent measurement, additional breast milk or infant formula was used for further extraction of lipids.

To measure the ratio of total PA to total fatty acids ([PA in total FA (%)]), 4 mg of lipid extract was used. Phospholipids in the lipid extracts were removed using solid-phase extraction (InertSep SI; GL Science). Lipids were methylated using a fatty acid methylation kit (nacalai tesque), followed by gas chromatography. To measure the sn-2 PA ratio in the total sn-2 fatty acids ([PA in sn-2 FA (%)]), 50 mg of lipid extract was used. After the removal of phospholipids in lipid extracts using a solid phase extraction (InertSep SI; GL science) technique, sn-1 and sn-3 fatty acids were digested using lipase (Chirazyme L-2 C4; Roche diagnosis) at 50 °C for 10 min, and 30 °C for 170 min. After the reaction, the free fatty acids were removed using a solid-phase extraction (InertSep SI; GL science) technique. The sn-2 monoacylglycerols were methylated using a fatty acid methylation kit (nacalai tesque), followed by gas chromatography.

Gas chromatography analysis was performed using a GC-2010 Plus (Shimadzu) coupled with a flame ionization detector. Separation was achieved on a ZB-FAME column (length=20 m, internal diameter=0.18 mm, film thickness=0.15 μm; phenomenex). The conditions for analysis were as follows: career gas, helium; total flow rate, 36.2 mL/min; column flow rate, 0.61 mL/min; and column temperature, 100−100 °C/0−2 min, 100−140 °C/2−6 min, 140−190 °C/6−23 min, 190−260 °C/23−25 min, and 260−260 °C/25−27 min. The [PA in total FA (%)] and [PA in sn-2 FA (%)] were calculated as the ratio of the peak area. The sn-2 PA bonding ratio ([sn-2 bond ratio of PA (%)]) was calculated using the following equation:

$$[\text{sn-2 bond ratio of PA (\%)}] = [\text{PA in sn-2 FA (\%)}]/(3 \times [\text{PA in total FA (\%)}])$$

Table S1. PA ratio in sn-2 position, PA ratio in total fat, and fat and energy content in breast milk at 1 month and infant formula available in Japan

Sample		sn-2 PA in total PA (%)	PA in total fat (%)	Fat (g/100 mL)	Energy (kcal/100 mL)	Category
Breast milk		73.9 ± 6.1 *[1]	22.6 ± 2.2 *[2]	3.6 ± 1.3 *[3]	69 ± 12 *[3]	-
Infant formula	A	55.3	20.6	3.5	68	High sn-2 PA milk
	B	52.9	20.2	3.5	68	
	C	39.5	22.0	3.6	66	Low sn-2 PA milk
	D	37.5	21.8	3.8	68	
	E	11.9	19.3	3.6	67	
	F	11.7	25.3	3.6	67	
	G	11.4	19.2	3.6	67	
	H	10.3	18.2	3.6	67	
	I	6.4	23.3	3.5	67	
	J	6.1	22.9	3.5	67	

*[1]: n=139, *[2]: n=140, *[3]: n=92

Abbreviations: sn, stereospecific numbering; PA, palmitic acid

Table S2. Results of stool characteristics

		Exclusively breastfed	Formula-fed		*p*-value *
			High sn-2 PA milk-fed	Low sn-2 PA milk-fed	
At 1 month					
Fecal Ca levels (mg/g-dry-stool)		18.3 ± 6.5 (n=12)	32.6 ± 17.1 (n=22)	28.0 ± 12.6 (n=45)	0.015 **
Fecal water levels (w/w%)		73.1 ± 8.2 (n=27)	72.4 ± 9.3 (n=31)	72.9 ± 9.9 (n=69)	0.953
Stool consistency *** (n)	Watery	2	4	15	0.172
	Soft	25	32	55	
	Formed	2	0	5	
	Hard	0	1	0	

*: One-way analysis of variance for continuous variable, or Chi-squared test for category number

**: *p*=0.011 (Exclusively breastfed vs High sn-2 PA milk-fed), *p*=0.074 (Exclusively breastfed vs Low sn-2 PA milk-fed), and *p*=0.385 (High sn-2 PA milk-fed vs Low sn-2 PA milk-fed) in Turkey-Kramer's test

***: Categorization was followed by a scale of Bekkali et al [3].

Abbreviations: sn, stereospecific numbering; PA, palmitic acid

Table S3. Association between feeding volume of high/mid/low sn-2 PA milk in the multiple regression analysis showing total PA levels in stool (mg/g-dry-stool).

	β	95%CI	*p*-value
Feeding volume of high sn-2 PA milk	0.01	−0.14–0.16	0.882
Feeding volume of mid sn-2 PA milk	0.30	0.14–0.46	$p < 0.001$
Feeding volume of low sn-2 PA milk	0.45	0.34–0.55	$p < 0.001$

The analytical model was adjusted by fecal pH

The unit of feeding volume of each infant formula is mL/day/kg

Abbreviations: sn, stereospecific numbering; PA, palmitic acids; β, the partial regression coefficient; 95%CI, the 95% confidence interval

Table S4. Association between feeding volume of high/mid/low sn-2 PA milk in the multiple regression analysis showing soaped PA levels in stool (mg/g-dry-stool).

	β	95%CI	*p*-value
Feeding volume of high sn-2 PA milk	−0.02	−0.16–0.12	0.799
Feeding volume of mid sn-2 PA milk	0.24	0.09–0.39	0.002
Feeding volume of low sn-2 PA milk	0.31	0.21–0.40	$p < 0.001$

The analytical model was adjusted by fecal pH

The unit of feeding volume of each infant formula is mL/day/kg

Abbreviations: sn, stereospecific numbering; PA, palmitic acids; β, the partial regression coefficient; 95%CI, the 95% confidence interval

References

1. Watanabe, Y.; Sato, S.; Asada, M.; Arishima, T.; Iida, Y.; Imagi, J.; Saito, K.; Sano, T.; Sasaki, A.; Sasaki, R.; et al. Enzymatic Analysis of Positional Fatty Acid Distributions in Triacylglycerols by 1(3)-Selective Transesterification with Candida antarctica Lipase B: a Collaborative Study. *J Oleo Sci.* **2015**, *64*, 1193-1205, doi:10.5650/jos.ess15182
2. Bligh, E.G.; Dyer, W.J. A rapid method of total lipid extraction and purification. *Can J Biochem Physiol.* **1959**, *37*, 911-917, doi:10.1139/o59-099
3. Bekkali, N.; Hamers, S.L.; Reitsma, J.B.; Van Toledo, L.; Benninga, M.A. Infant stool form scale: development and results. *J Pediatr.* **2009**, *154*, 521-526.e521, doi:10.1016/j.jpeds.2008.10.010
